# Farmland increases Indian crested porcupine occupancy in Parsa-Koshi complex, Nepal

**DOI:** 10.1371/journal.pone.0315307

**Published:** 2024-12-31

**Authors:** Bishal Subedi, Sandeep Regmi, Bishnu Prasad Bhattarai, Hem Bahadur Katuwal, Ashok Kumar Ram, Jerrold L. Belant, Hari Prasad Sharma

**Affiliations:** 1 Central Department of Zoology, Institute of Science and Technology, Tribhuvan University, Kirtipur, Kathmandu, Nepal; 2 Center for Integrative Conservation, Xishuangbanna Tropical Botanical Garden, Chinese Academy of Sciences, Mengla, Yunnan, China; 3 Nepal Zoological Society, Kirtipur, Kathmandu, Nepal; 4 Department of National Parks and Wildlife Conservation, Kathmandu, Nepal; 5 Department of Fisheries and Wildlife, Michigan State University, East Lansing, MI, United States of America; Universidade Federal do Para, BRAZIL

## Abstract

Understanding species distributions and factors influencing them are important for conservation, particularly for species occurring in human-dominated areas. The Indian crested porcupine (*Hystrix indica*; hereafter porcupine) is distributed southeast and central Asia, however, the porcupine occurrence and habitat use is poorly understood in the area. We deployed cameras at 154 sites for 21 days (3234 trap nights) during December 2022–March 2023 in the human-dominated landscape of Parsa-Koshi Complex (PKC), Madesh Province, Nepal. We used single season single species occupancy model to estimate the relationship of selected covariates with porcupine occupancy. We identified moderate occupancy [0.321 ± 0.079 (SD)] and detection probability [0.315 ± 0.076 (SD)] of porcupines. Although porcupine occurrence was greater in protected area than in outside protected areas, occupancy was positively associated with area of farmland (1.531 ± 1.703) and human presence (0.459 ± 0.531), while it declined with increasing forest canopy cover (-0.86 ± 0.363). The positive effects of agricultural areas and human presence demonstrate the adaptability of porcupines to humans and the potential for continued conflicts. Based on these baseline data, policy makers and wildlife managers can gain insight into the pattern of porcupine occurrence and aid targeted conservation strategies to mitigate increasing human-porcupine conflicts in PKC.

## Introduction

Understanding how various factors influence species distribution is important for their conservation and facilitating human-wildlife coexistence [1,2]. Resource availability, natural habitat attributes, and human activities like habitat disturbance can markedly influence species occurrence [3,4]. However, the influence of these factors varied across spatial and temporal scales [5,6], and are more pronounced in burrowing animals [7].

The Indian crested porcupine (*Hystrix indica*; hereafter porcupine) is a burrowing animal that distributes southeast and central Asia and in some parts of the Middle East [8,9]. Porcupine use various habitats such as temperate scrublands, grassland, forest, and plantations [10,11], as well as croplands near forest edges [12,13]. Habitat use of porcupines can be influenced by tree height, soil type, distance to farmland [7], proximity to human settlements [14], and presence of large predators such as tiger and leopard [15]. As an herbivore, the porcupine thrives in a human-dominated landscape, with farmland providing both abundant food and shelter [16,17]. Although large predators like tigers (*Panthera tigris*) and leopards (*P*. *pardus*) could affect porcupine behavior through predation risk [15]. Predator avoidance is not a primary driver of porcupine activity. Instead, these predators have adapted their activity pattern to target their main prey such as ungulates, with little evidence of significant predation on the porcupine [18,19].

Porcupines are known to cause substantial damage to agricultural crops, which is a significant source of conflict between humans and wildlife, particularly in and around protected areas. [12,20–22]. In region such as Pakistan and India, Porcupines have been reported to feed on both subsistence and commercial crops, causing significant agricultural losses[23,24]. In Nepal, this issue is especially pronounced near protected areas like Banke National Park, Kanchenjunga Conservation Area, Khaptad National Park, Chitwan National Park [25–28], where the economic damage is considerable. Outside protected areas, the mid-mountain regions have experienced the greatest economic loss, with an estimated total annual damage of NPR 4,33,137 (US $ 3670) [29]. This crop depredation often brings porcupines into close proximity to human settlements, increasing the likelihood of conflict. Despite these significant impacts, limited information on the extent of human-porcupine conflicts [26,27,30] and species habitat preferences and distribution patterns [10,15]. A comprehensive understanding of porcupine occurrence and distribution across Nepal, including within Parsa-Koshi Complex (PKC) is lacking.

Due to the potential conflict between humans and porcupines as a results of crop damages by the species, effective mitigation of human-porcupine conflicts in Nepal is challenging and underscores the need to understand factors influencing porcupine occurrence and distribution. We aimed to address this knowledge gap in PKC, Nepal, focusing on porcupine associations with anthropogenic and ecological variables, such as presence of large predators, area of croplands, canopy cover, and presence of human in human dominated landscapes. The predominant crops in the study area were mainly rice (*Oryza sativa*), maize (*Zea mays*), sugarcane (*Saccharum officinarum*), and wheat (*Triticum aestivum*), and we measured variables as area coverage to quantify the effect of the agricultural factors on porcupine occurrence. Thus, we aimed to assess the occupancy of porcupine as well as understand the effects of environmental and anthropogenic factors on the porcupine occupancy in PKC. We hypothesized that porcupine occurrence would increase in areas closer to croplands and human presence, while decreasing in areas with higher canopy cover. By examining these relationships, we can gain insights into the factors affecting porcupine occurrence and aid conservation efforts to reduce increasing human-porcupine conflicts in PKC.

## Materials and methods

### Study area

**Fig 1:**
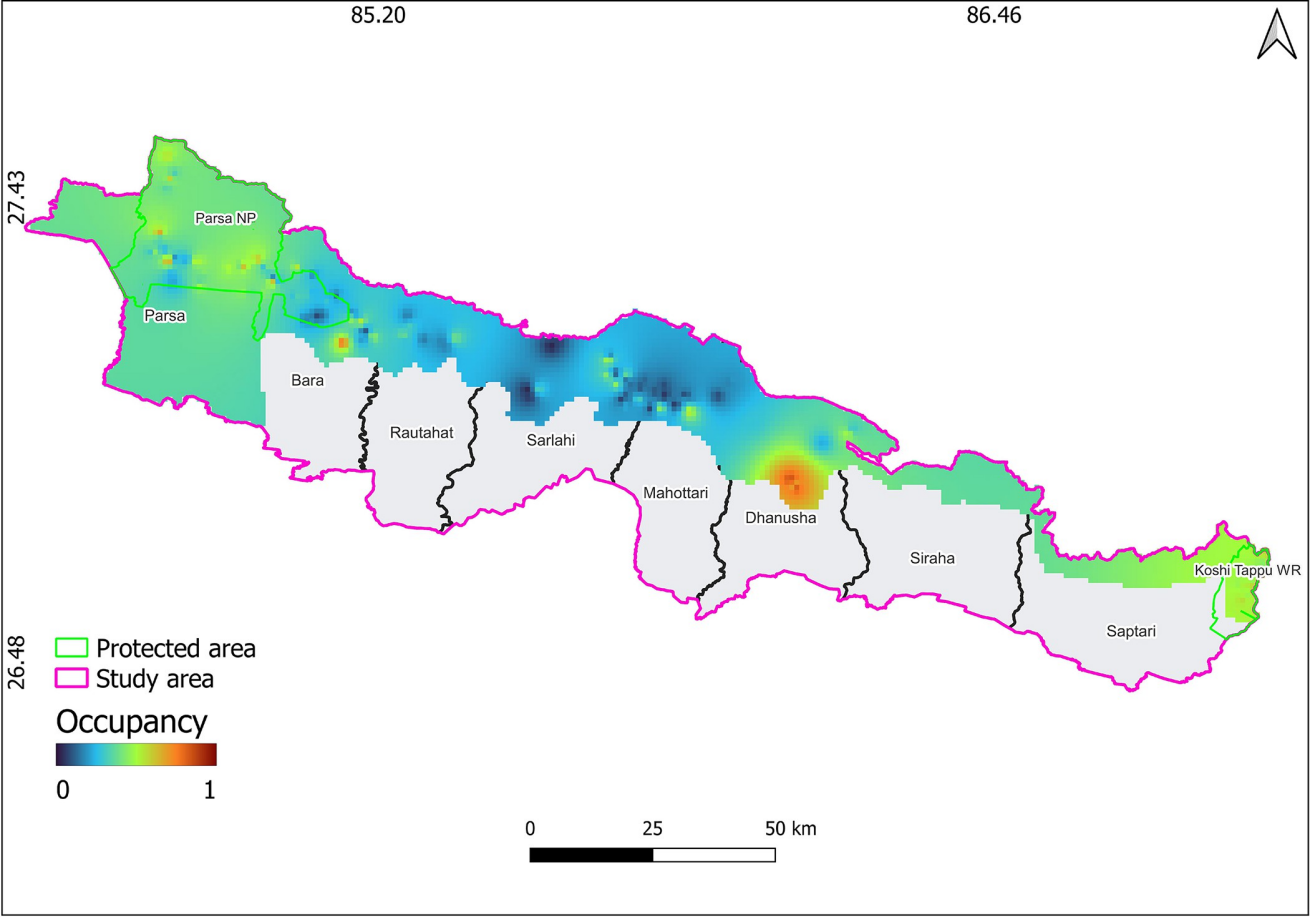
Study area of porcupine, Parsa- Koshi Complex, Nepal, December 2023–March 2023. The map is licensed under a Creative Commons by Attribution (CC BY 4.0) [41].

We conducted the study in Parsa-Koshi Complex (PKC), Nepal (Fig 1). The PKC comprises 9661 km^2^ with an elevational range of 80–800 m above sea level. The PKC lies in Madhesh Province of Nepal, ranges from Parsa National Park in the west to Koshi Tappu Wildlife Reserve in the east. The landscape contains various community forests, religious forests, and a forest corridor in the lowland regions [31–35]. Approximately 64.3% of the people in and around PKC rely on crop such as rice, wheat, sugarcane, corn, potatoes (*Solanum tuberosum*) and other vegetables are major crops produced and livestock agriculture for their livelihoods as well as forest products including firewood, leaves, and timber [36]. Primary vegetation types of the area are tropical and subtropical forests dominated by Sal (*Shorea rubusta*) and mixed forests dominated by Khair (*Acacia catechu*). These forests support more than 50 mammalian species including the porcupine, tiger, leopard, dhole, sloth bear (*Melursus ursinus*), Asiatic elephant (*Elephas maximus*), and greater one-horned rhino (*Rhinoceros unicornis*) [10,37–40].

### Data collection

We collected porcupine presence locations during December 2022–March 2023 using trail cameras. We deployed cameras at 154 sites for 21 days (3234 total trap nights) throughout PKC, maintaining a minimum distance of 1 km between cameras. We placed cameras 40–60 cm above ground along trails used by porcupine. For data collection including camera placement and camera setup, we followed [42]. We programmed cameras to take 3 images each detection with a 30-sec delay between detections.

At each camera trap station, we recorded habitat variables, such as canopy cover, the number of humans detected, presence of large predators, the nearest human settlement, and major road. Further, the species being a crop raider, we also took the area of farmland within 500 m radius of trap points using ESRI Sentinel-2 land-use land-cover map at a 10 m resolution [41]. A 10 m × 10 m plot was formed centering the camera trap station and canopy cover was calculated as the average of the four corners and the center using Gap Light Analysis Mobile Application[43]. The data on the number of humans and livestock detected was taken from the same camera traps. Area of farmland was measured using QGIS. The distance to the nearest permanent water body, distance to the nearest human settlement, and distance to the nearest major road were measured using measuring tape, but whenever the distance exceeded 200 m, it was measured using NNJOIN tool in QGIS.

### Ethical considerations for camera trap studies

Camera traps research permission was obtained from the Department of Forest and Soil Conservation (Permission Number: 596) and the Department of National Parks and Wildlife Conservation (Permission Number: 1165). We informed people on use of camera before deploying.

### Data analysis

We processed and analyzed the data in the R programming environment (R Core Team, 2023) using hierarchical occupancy modeling [44] to assess detection probability, occupancy, and impacts of covariates on porcupines. The detection probability refers to the likelihood of detecting a particular species across multiple sites, while occupancy represents the proportion of sampling units where the target species is present [45]. We formed the object data by organizing porcupine detections at each site *i* into a matrix. This matrix included the number of sampling replicates with species detections, with every 7 days of the 21-day camera trap deployment grouped into one sampling occasion, resulting in 3 sampling occasions per site. We standardized all predictor variables to account for differences in scale (e.g., road distance, number of human, canopy cover). Further, we used the detection probability of only two large carnivores; tiger and leopard collectively as the variable of presence of large predators. The variables used for the study were assessed for multicollinearity using a threshold of |r| > .7; no predictors were highly correlated, and we included all predictors in analysis.

We used occupancy as a measure of habitat selection instead of true occupancy estimate [46]. We derived occupancy as:

logit (ψi) = β0 + βs_ettlement_settlement_i_+ β_livestock_livestock_i_ + β_predators_predatorsi + β_cropland_cropland_i_ + β_canopycover_canopycover_i_ + β_human_human_i_ + β_road_road_i_

Where, *β*_*0 =*_ *logit*(*ψ*_*0*_) is the occupancy probability on the logit scale with zero value for the predictors, *βs*_*ettlement*_*settlement*_*i*_ is the impact of settlements on porcupines at site *I*, *β*_*livestock*_*livestock*_*i*_ is the impacts of livestock occurrence, *β*_*crops*_*crops*_*i*_ is the impact of crops area, *β*_*canopycover*_*canopycover*_*i*_ is the impacts of canopy cover, *β*_*human*_*human*_*i*_ is the impacts of human presence, and *β*_*roads*_*roads*_*i*_ is the impacts of distance to nearest road on porcupine occurrence.

We generated model output using Markov Chain Monte Carlo (MCMC) simulations, and confirmed model convergence by evaluating the Rhat value, using a threshold of 1.1. MCMC is a Bayesian probabilistic machine learning method capable of sampling probability distributions [47]. We ran the adaptive MCMC simulation using the packages jagsUI [48], and coda [49] in R program and Just Another Gibbs Sampler (JAGS); [50]. The analysis was done with three chains, 1000 burnins, 1000 adaptations, and 10,000 iterations. Further, we used Inverse Distance Weighting (IDW), a spatial interpolation technique, to estimate occupancy probabilities across unsampled locations. IDW calculates weighted averages of nearby points, with closer points having greater influence on the estimate [51].

## Results

**Fig 2:**
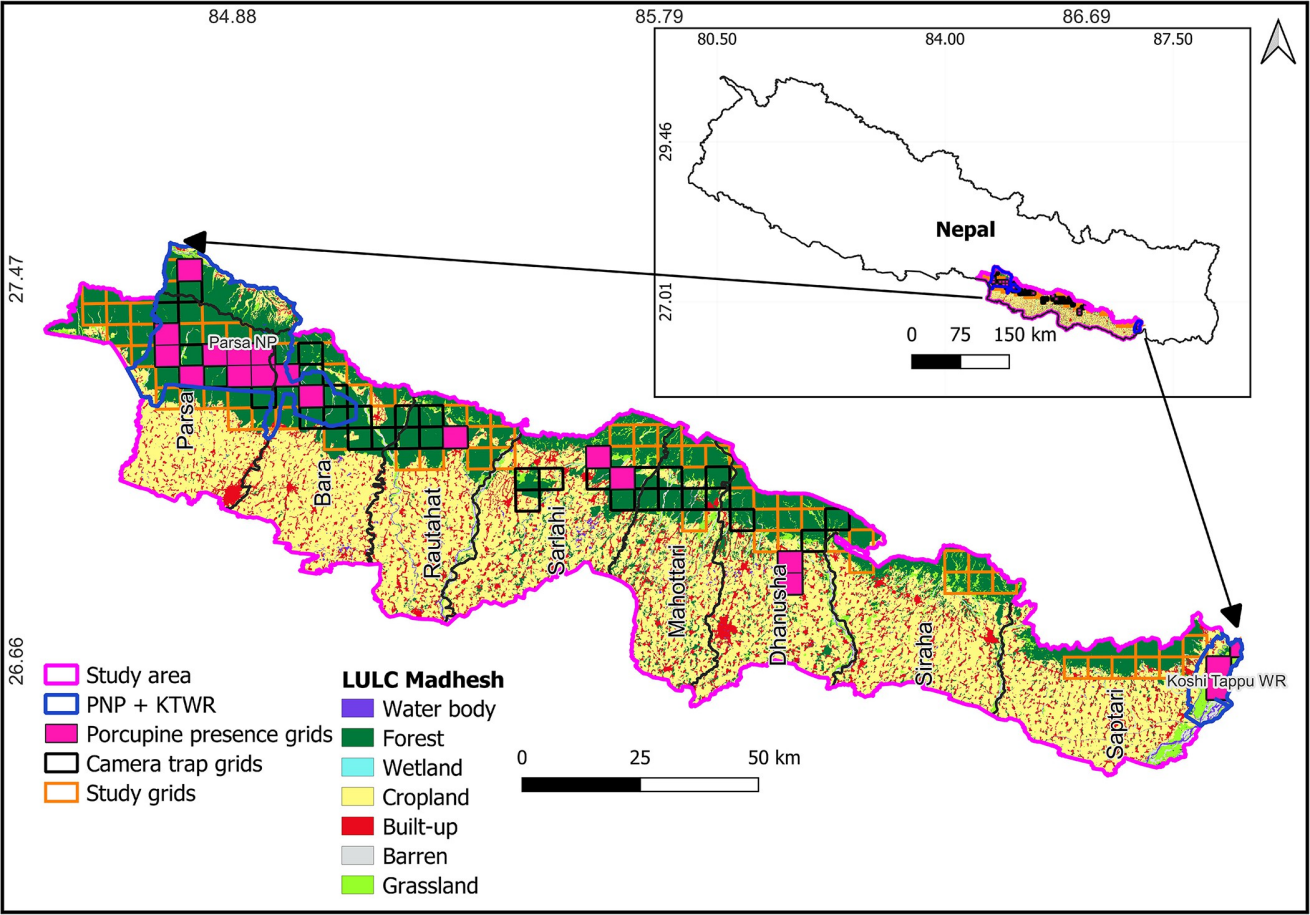
Estimated effect size of covariates on porcupine occupancy with upper and lower credible intervals, Parsa–Koshi Complex, Nepal December 2023–March 2023.

Overall, we detected porcupines at 29 of 154 sites, which was greater in protected areas. We found that porcupine had an occupancy rate of 0.338 ± 0.137 (mean ± SE) and detection probability of 0.308 ± 0.076 (mean ± SE) (S1 Table). Mean canopy cover was 42.19 ± 21.36 (SD)%; mean numbers of human and livestock detections were 76.72 ± 244.55 (SD) and 36.74 ± 102.45 (SD), respectively. Mean distance to the nearest road was 741.69 ± 1138.91 (SD) m, and mean distance to the nearest settlement was 2182.80 ± 1691.33 (SD) m. Further, there was a significant positive impact of the area of farmland (*βcropland* = 1.856 ± 1.965) and the number of human presence (*βhuman* = 0.507 ± 0.677) on occupancy of porcupine where area of farmland exerted a greater impact (Fig 2). There was a negative association between porcupine occupancy and canopy cover (*βcanopycover* = -1.008 ± 0.373) (Fig 2). Conversely, no significant relationship was observed between porcupine occupancy and distance to road (*βroad* = 1.999 ± 1.735), settlements (*βsettlements* = 0.028 ± 0.421), and number of livestock (*βlivestock =* 0.199 ± 0.443) or large predators (*βpredators =* 0.362 ± 0.308) (Fig 2).

**Fig 3:**
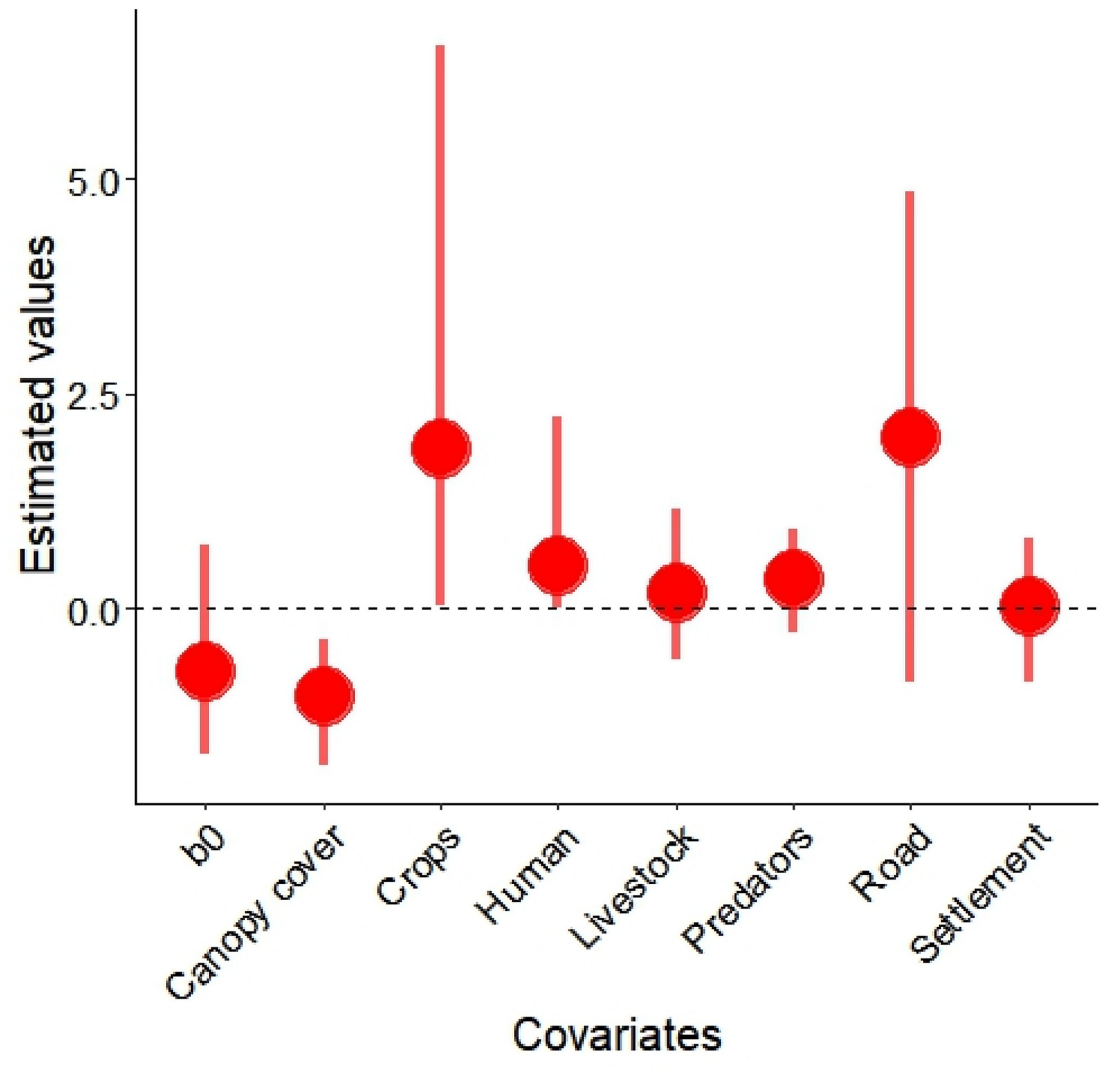
Inverse distance weighting (IDW) interpolation based on occupancy probability map of porcupine, Parsa-Koshi Complex, Nepal, December 2023–March 2023. The map is licensed under a Creative Commons by Attribution (CC BY 4.0) [41].

We observed higher occupancy probability of porcupine in Parsa followed by Dhanusha, and Bara regions (Fig 3). There was limited probability of occupancy in Mahottari, Sarlahi and Saptari. However, Siraha had relatively low occupancy probability for porcupines (Fig 3).

## Discussion

Occupancy and detection probability of porcupines in a significant portion of the study area appeared influenced by farmland area, dense canopy cover and human presence. The detection probability of porcupines was greater inside the protected areas than outside, potentially due to superior habitat quality, reduced anthropogenic pressure and availability of food [4,16,17]. We observed an increase in porcupine occupancy with increasing area of farmland which might be due to porcupine dependency on agricultural crops [9,23,52–55], which suggests that porcupines may utilize agricultural areas for foraging but prefer forest habitats for shelter and resources. Similarly, occurrence was also found to be positively associated with farmland in Rome, Italy [56]. Porcupines may exploit nearby cultivated areas with easy access to food from crops (e.g., rice, potatoes), requiring less time for excavating roots and tubers [12]. Rice, wheat, sugarcane, corn, potatoes and other vegetables are major crops produced in PKC [57] which are severely damaged by porcupine in irrigated plains and other areas [10,26,52,53,57]. Extensive cultivated fields may offer better habitat for porcupines if they are interspersed with natural or semi-natural vegetation areas, which would provide shelter in addition to abundant food [58–60].

The positive association between porcupine occurrence and the number of humans detected might be due to porcupine use of plantations along canals and intensively cultivated croplands [61–63]. Presence of humans is typically high near the forest edge and within plantations along canals due to resource collection [38], as humans and porcupines share similar foods [57]. This co-occurrence could pose risks to porcupines [64].

We observed a negative association between porcupine occurrence and canopy cover. Dense canopy cover might limit food availability [65] and inhibit sunlight penetration, reducing growth of understory plants [66] that porcupines consume [17,52]. A similar pattern was observed by Luzi et al., [67] where porcupines selected forests with intermediate canopy cover composed of mixed forests with extensive edge. We found no significant association of porcupine occupancy with distance to major roads and livestock. Porcupines prefer habitat within their territory where resources are abundant and provide shelter [14] which are typically unavailable near roads. Similarly, the non-significant effect of livestock on porcupine occupancy might be due to variables unexplored in our study and requires further evaluation.

We note several potential limitations in our study. First, our study was restricted to winter, potentially overlooking seasonal variations in porcupine behavior and occupancy pattern. Additionally, our survey was constrained to lower elevations (<300 m) of PKC due to the challenges in accessing the Chure Region. Moreover, the presence of high human disturbance, particularly related to resources collection and livestock grazing, led to loss of some cameras due to vandalism. Despite these limitations, our study provides knowledge of factors influencing porcupine occupancy which can be applied to management of porcupine populations in human-dominated landscapes.

## Conclusions

Our study provides insights on the occupancy of porcupine in PKC and how it changes with anthropogenic and ecological covariates. Porcupines exhibited a moderate detection probability, suggesting detecting this species within this area can be challenging. Further, the positive relationships between porcupine, farmland, and human presence, underscore the species’ adaptability to anthropogenically altered landscapes and its resilience to fragmented habitat. However, such co-occurrence may pose serious threats like retaliatory killing and illegal hunting of the species which highlights the necessity to develop effective conservation strategies to balance ecological and socio-economic interchange between humans and porcupines. We recommend implementing proper fencing and compensation programs to enhance the human-porcupine co-existence in the PKC region.

## Supporting information

S1 Table Indian crested porcupine occupancy and covariate estimates, Parsa Koshi Complex, Nepal, December 2023–March 2023. P = detection probability, psi = naïve occupancy, β = occupancy in logit scale for variables, SD = standard deviation, LCI = lower credible interval, UCI = upper credible interval, Rhat = ratio of the variance of a parameter, n.eff = effective sample size, overlap0 = proportion of posterior with same size, and f = f statistics. Significant effects in bold type.
(DOCX)
