# Correction: Farmland increases Indian crested porcupine occupancy in Parsa-Koshi complex, Nepal

**DOI:** 10.1371/journal.pone.0329661

**Published:** 2025-08-04

**Authors:** Bishal Subedi, Sandeep Regmi, Bishnu Prasad Bhattarai, Hem Bahadur Katuwal, Ashok Kumar Ram, Jerrold L. Belant, Hari Prasad Sharma

The images for Figs 1–3 are incorrectly switched. The image that appears as Fig 1 should be Fig 3, Fig 2 should be Fig 1 and the image that appears as Fig 3 should be Fig 2. The figure captions appear in the correct order. The authors have provided a corrected version of figures here.

**Fig 1:**
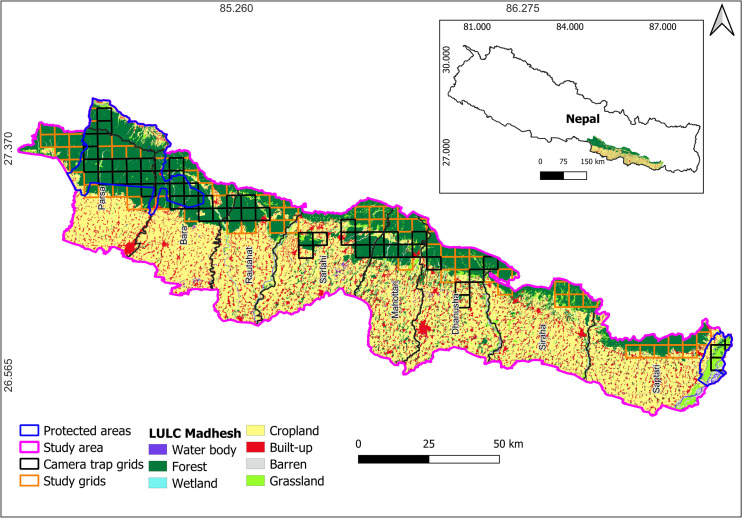
Study area of porcupine, Parsa- Koshi Complex, Nepal, December 2023–March 2023. The map is licensed under a Creative Commons by Attribution (CC BY 4.0) [41].

**Fig 2:**
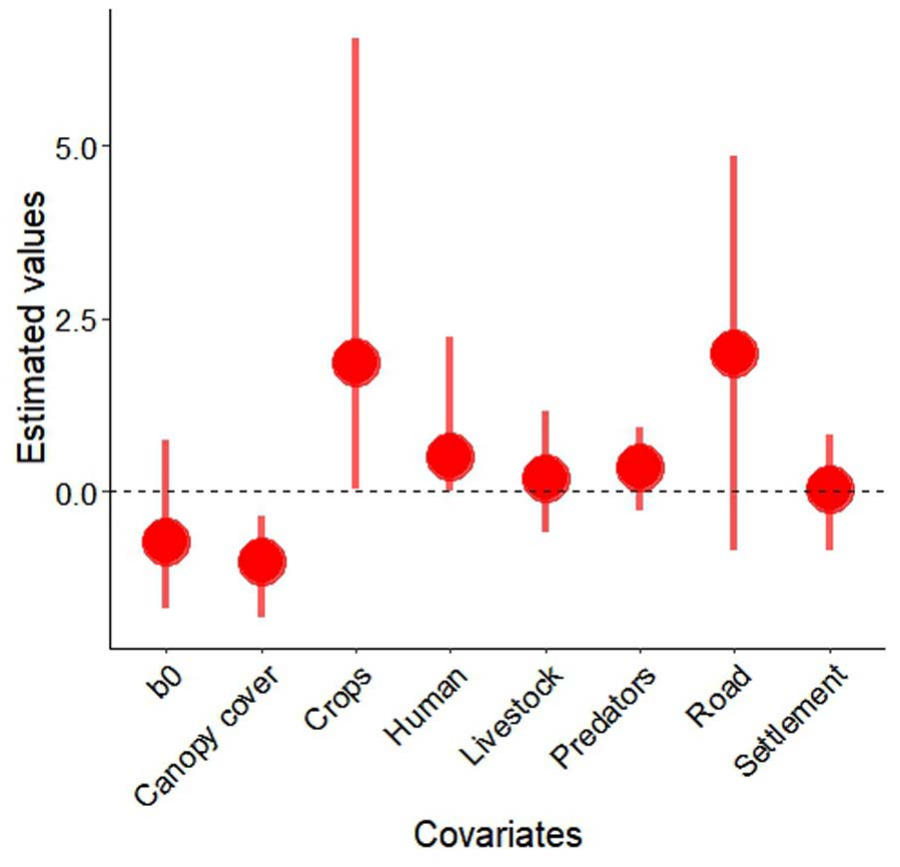
Estimated effect size of covariates on porcupine occupancy with upper and lower credible intervals, Parsa–Koshi Complex, Nepal December 2023–March 2023.

**Fig 3:**
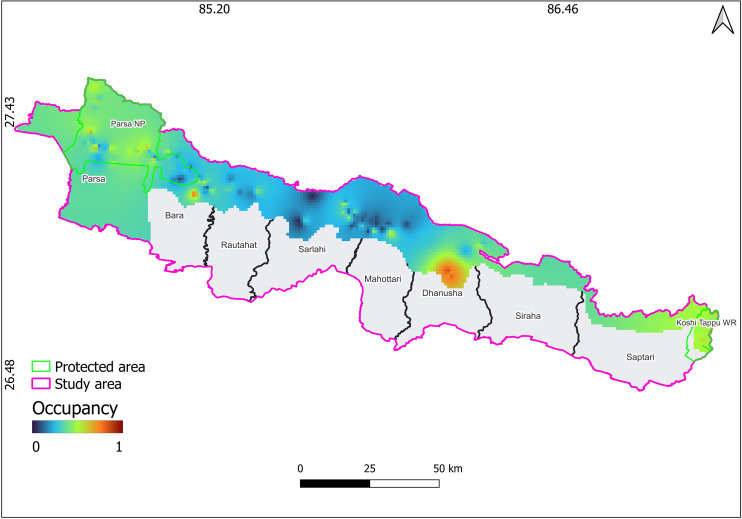
Inverse distance weighting (IDW) interpolation based on occupancy probability map of porcupine, Parsa-Koshi Complex, Nepal, December 2023–March 2023. The map is licensed under a Creative Commons by Attribution (CC BY 4.0) [41].
