# Supplementary material for: Farmland increases Indian crested porcupine occupancy in Parsa-Koshi complex, Nepal
Source: PLoS One. 2024 Dec 31;19(12):e0315307. doi: 10.1371/journal.pone.0315307 (PMC11687721; doi:10.1371/journal.pone.0315307)
Supplement: S1 Table — P = detection probability, psi = naïve occupancy, β = occupancy in logit scale for variables, SD = standard deviation, LCI = lower credible interval, UCI = upper credible interval, Rhat = ratio of the variance of a parameter, n.eff = effective sample size, overlap0 = proportion of posterior with same size, and f = f statistics. Significant effects in bold type. (DOCX) [file pone.0315307.s001.docx]

**S1 Table 1. Indian crested porcupine occupancy and covariate estimates, Parsa Koshi Complex, Nepal, December 2023–March 2023.** P= detection probability, psi= naïve occupancy, β = occupancy in logit scale for variables, SD = standard deviation, LCI = lower credible interval, UCI = upper credible interval, Rhat = ratio of the variance of a parameter, n.eff = effective sample size, overlap0 = proportion of posterior with same size, and f = f statistics. Significant effects in bold type.

| Parameters | mean | sd | LCI | UCI | Rhat | overlap0 |
| --- | --- | --- | --- | --- | --- | --- |
| P | 0.308 | 0.076 | 0.172 | 0.467 | 1.002 | 0 |
| Psi | 0.339 | 0.137 | 0.155 | 0.677 | 1.008 | 0 |
| β0 | -0.717 | 0.634 | -1.692 | 0.738 | 1.008 | 1 |
| **Canopy cover (%)** | **-1.009** | **0.373** | **-1.830** | **-0.356** | **1.001** | **0** |
| **Farmland area** | **1.857** | **1.965** | **0.033** | **6.546** | **1.003** | **0** |
| Livestock | 0.199 | 0.443 | -0.596 | 1.154 | 1.004 | 1 |
| **Human (n)** | **0.507** | **0.678** | **0.008** | **2.231** | **1.025** | **0** |
| Distance to road (m) | 1.999 | 1.735 | -0.852 | 4.851 | 1.000 | 1 |
| Distance to settlement (m) | 0.028 | 0.422 | -0.853 | 0.813 | 1.003 | 1 |
| Presence of Large predators | 0.362 | 0.308 | -0.269 | 0.919 | 1.001 | 1 |
